# Surgical treatment of primary solitary fibrous tumors involving the pelvic ring

**DOI:** 10.1371/journal.pone.0207581

**Published:** 2018-11-27

**Authors:** Yifei Wang, Ran Wei, Tao Ji, Zhongyan Chen, Wei Guo

**Affiliations:** Department of Musculoskeletal Tumors, Peking University People’s Hospital, Beijing, People’s Republic of China; University of Manitoba, CANADA

## Abstract

The surgical treatment of primary solitary fibrous tumors (SFT) involving the pelvic ring has not been reported previously. In this study, we aimed to evaluate the efficacy of surgical treatment for this disease. From 2009 to 2015, 13 patients underwent tumor resection at our hospital, with an average age of 49.5 years (27–68 years). Four patients underwent en bloc resection, and 9 patients underwent piecemeal resections. A Chi-square test was used to compare the postoperative recurrence rates between the en bloc resection and the piecemeal resection groups (*p* = 0.68), and no significant difference was found between the benign and malignant solitary fibrous tumor groups (*p* = 0.44). The 5-year survival rate of the patients in this study was 83.3%, and the 5-year progression-free survival rate was 63.5%. The progression-free survival rate was not significantly different between the en bloc resection and piecemeal resection groups (*p* = 0.97). Piecemeal resection can also achieve acceptable local control, particularly for patients with sacral tumors, as they may achieve even better postoperative function with sacral nerve preservation. Recurrence and metastasis occur relatively late in the course of this disease. Therefore, long-term follow-up is required.

## Introduction

Solitary fibrous tumors are a type of rare fibroblastic tumor originating from the mesenchyme and accounting for less than 2% of all soft tissue tumors[1]; the annual incidence of these tumors is only two per one million tumors[2]. Solitary fibrous tumors are commonly seen in the pleura but can also occur in the limbs, pelvic cavity, abdominal cavity, neck, and central nervous system. However, solitary fibrous tumors with bone involvement are extremely rare and have not been reported separately in previous studies.

Surgical treatment is the preferred treatment method for this disease[3–5]. However, the disease course of tumors involving the pelvic ring is relatively insidious; therefore, the tumors are generally larger in volume at the time of diagnosis, which results in difficult surgical resection, a large bleeding volume, significant postoperative motor dysfunctions, and urinary and bowel incontinence. Therefore, a reasonable surgical treatment method should be selected that can achieve satisfactory local control while retaining patient functions. To the best of our knowledge, this study includes the largest number of cases of primary solitary fibrous tumors involving the pelvic ring to date.

## Data and methods

### General data and statistical methods

The study is approved by the institutional review board of Peking University People's Hospital. All data were anonymized before analyzed by the researchers. And the IRB waived the requirement for informed consent. From March 2009 to October 2015, 13 patients with primary solitary fibrous tumors involving the pelvic ring underwent tumor resection at our hospital were included in this study. Of them, 5 were male, and 8 were female, with an average age of 49.5 years (27–68 years). The average follow-up time was 43.2 months (15–84 months). Innominate bone involvement was observed in 6 patients, and sacral involvement was observed in 8 patients (Table 1). Among all the patients, 2 had experienced recurrence after surgery at other hospitals, while all other patients started their initial treatments at our hospital. Among the 8 patients with sacral tumors, 4 (50%) reported local pain in the sacrococcygeal area at the time their hospital visit, 5 (62.5%) exhibited neurological symptoms in the lower extremities, and 3 (37.5%) experienced urinary and bowel incontinence. Among the 5 pelvic patients, all 5 (100%) reported local pain at the time of visiting the hospital, 4 (80%) exhibited neurological symptoms in the lower extremities, and 1 experienced urinary and bowel incontinence (12.5%). One patient, whose tumor involved both the innominate bone and the sacrum, reported radiating pain and local pain in the lower extremities. Patients with solitary fibrous tumors in the region without bone involvement were excluded from this study.

**Table 1:** Summary of the clinical data of 13 patients with primary solitary fibrous tumors.

No	Sex	Age	Location	Resection method	Follow-up result	Recurrence and metastasis	Malignancy status	Other treatment
1	Female	51	Innominate bone (region I)	En bloc	DOD	Bone metastasis		
2	Female	63	Sacrum (S1-2)	Piecemeal	AWD	Recurrence, Bone metastasis		Radiotherapy after recurrence
3	Male	68	Innominate bone (region II)	Piecemeal	NED	Recurrence		En bloc resection after recurrence
4	Female	52	Sacrum (S1-2)	Piecemeal	NED	None		
5	Female	30	Sacrum (S3 and below)	Piecemeal	NED	None	Malignant	
6	Female	46	Sacrum (S1-2) Innominate bone (regions I+IV)	Piecemeal	NED	None	Malignant	
7	Male	42	Innominate bone (regions II+III)	En bloc	NED	None	Malignant	
8	Female	65	Innominate bone (regions I+II)	En bloc	NED	None		
9	Male	45	Sacrum (S2-3)	Piecemeal (subtotal resection)	AWD	Recurrence, Abdominal cavity metastasis	Malignant	Radiotherapy after recurrence
10	Female	27	Innominate bone (regions I+IV)	En bloc	NED	None	Malignant	
11	Female	48	Sacrum (S2-3)	En bloc	NED	None		
12	Male	68	Sacrum (S2-3)	En bloc	AWD	Abdominal cavity metastasis	Malignant	
13	Male	38	Sacrum (S3 and below)	Piecemeal	NED	None	Malignant	Preoperative use of apatinib

At the time of the hospital visit, all patients received preoperative plain X-ray, computed tomography (CT), and magnetic resonance imaging (MRI) examinations to determine the size and involvement of the tumor. Thoracic, abdominal, and pelvic CT and bone scan or positron emission tomographic (PET)-CT were performed to assess the general condition of the whole body. During surgery, none of the patients exhibited distant metastasis. For patients seeking an initial diagnosis, a preliminary diagnosis was determined according to the patient’s medical history data and the characteristics of the imaging studies. All patients were pathologically diagnosed through core needle or incision biopsy. The histological criteria for malignant solitary fibrous tumor are: more than 4 mitotic figures per 10 high-power fields, presence of necrosis or hemorrhage, increased cellularity, nuclear pleomorphism and stromal or vascular invasion. The average solitary fibrous tumor diameter was 11.5 cm; the smallest was 5 cm, and the largest was 23 cm.

A Chi-square test was used to compare the postoperative recurrence rates between the en bloc resection and piecemeal resection groups, and the Kaplan-Meier method was used to calculate the overall survival rate and to compare the event-free survival rates between the en bloc resection and piecemeal resection groups.

### Surgical methods

Resection of sacral solitary fibrous tumors: the posterior approach was mainly adopted. For the tumors involving S3 and below, upon entering the presacral space by removal of the sacrotuberous ligament and coccyx, the rectum was bluntly separated from the tumor, pushed forward, and then separated upward to approximately the S1 level. The sacrococcygeal bone was removed below S3. The lamina was opened posteriorly to expose the sacral canal, carefully separating the sacral nerve, and the tumor was removed from inside the sacral canal in an attempt to retain the bilateral nerve root above S3. The tumor in front of the sacrum was completely separated. For the tumors involving S2 and above, the surgery is also performed through a posterior approach. The sacrococcygeal bone was removed below S3. After the lamina was opened posteriorly, the dural sac and the S1-S3 nerves were identified and protected. The tumor invading the sacral canal was removed. To separate the tumor, a thick thread was used to pass through the tumor to pull and suspend the tumor. Upon mobilizing the tumor, gauze was packed into the presacral space to bluntly separate the rectum and sacral nerves from the tumor capsule. After the rectum and sacral nerves anterior to the tumor was protected. The anterior part of the sacrum including the tumor was resected from behind.

Resection of solitary fibrous tumors involving the innominate bone: According to the region of the tumor, en bloc resection was mainly used for tumors involving regions I or I+IV, and a pedicle screw and rod system was used for reconstruction of the defect. Four pedicle screws were fixed in the pedicle of the lumbosacral vertebrae, the pubis, and the ischium at the top of the acetabulum. Then, two rods were connected to maintain the integrity of the pelvic ring, a titanium cage was placed in the location with the bone defect, and bone grafting was performed.

For tumors involving region II, an internal hemipelvectomy method was frequently used. The incision used was an iliac groin incision and an auxiliary incision from the anterior superior iliac spine to greater trochanter. After separation of the retroperitoneal space, the vascular nerve bundles were first exposed, an abdominal aorta balloon block and/or ligation of the ipsilateral internal iliac artery was used to control bleeding. After the tumor was exposed, a wire saw was pulled through the greater sciatic foramen, and osteotomy was performed according to tumor involvement. After region II and region II+III tumors were resected, a modular hemipelvic prosthesis was used for reconstruction. During the simultaneous resection of region I and/or region IV tumors, pedicle screw and rod-type prosthetic hemipelvic replacement or autologous bone graft, pedicle screw and rod internal fixation, titanium mesh cup + total hip arthroplasty, or other reconstruction methods were used.

Solitary fibrous tumors often have abundant blood supplies; at present, we use an intra-abdominal aorta balloon block to temporarily control bleeding in all patients. Some patients also received preoperative embolization.

## Results

Four patients underwent en bloc resection, and 9 patients underwent piecemeal resection. Of these patients, 12 underwent gross total resection, and 1 patient underwent subtotal resection due to severe adhesion between the tumor and the pelvic organs. One patient received apatinib as a neoadjuvant therapy. Six patients with sacral tumors or the tumors involving innominate bone region II (2 patients) received preoperative embolization. Intra-abdominal aorta balloon block to temporarily control bleeding is used in all patients; the average bleeding volume of patients in this study was 1757 ml (400–6600 ml), and the average operation time was 4.2 h (3–6 h). No serious perioperative complications occurred. Three patients showed poor wound healing, 2 patients healed after dressing, and 1 patient healed after debridement and stitching. Seven patients were confirmed to have low-degree malignant solitary fibrous tumors. One patient died 32 months after surgery, Among the patients who received gross total resection, 2 patients with benign SFT who received piecemeal resection (2/12, 16.7%) experienced recurrence at the 16^th^ and 26^th^ postoperative months. One patient (malignant SFT) who received subtotal resection experienced recurrence at the 48^th^ month (the overall recurrence rate was 23.1%), and 3 patients exhibited metastasis at the 27^th^, 48^th^ (malignant SFT with piecemeal resection) and 72^nd^ (malignant SFT with en bloc resection) postoperative months. All patients reported normal urinary and bowel continence postoperatively.

**Fig 1:**
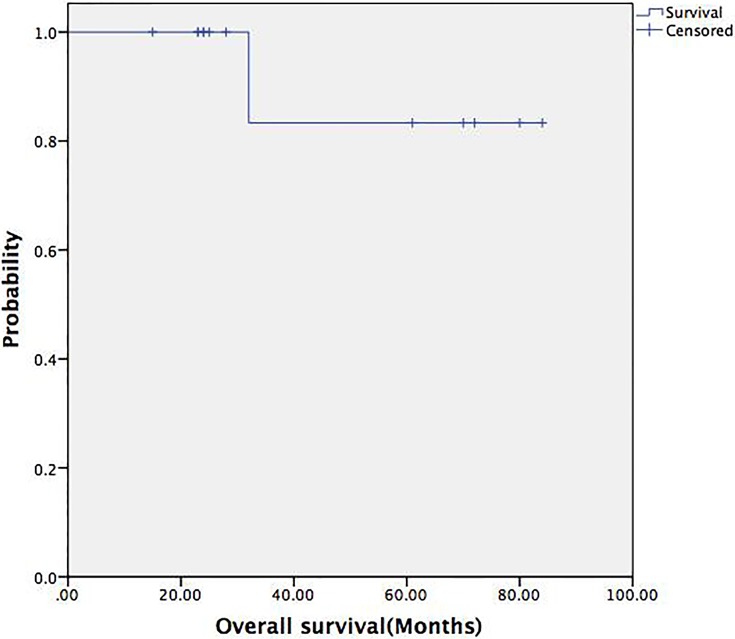
Overall survival curve of the 13 patients. The 5-year survival rate was 83.3%.

**Fig 2:**
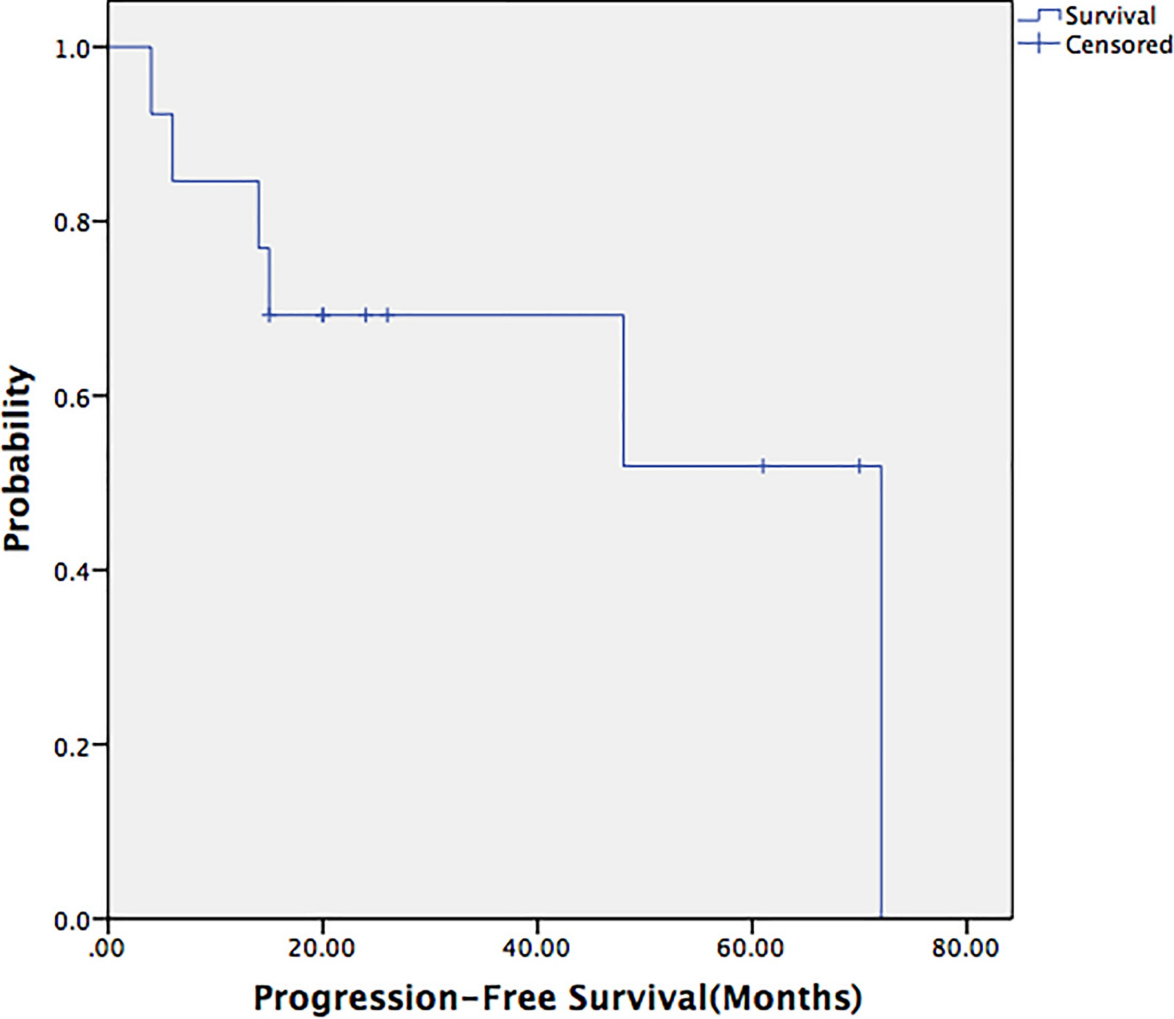
Progression-free survival curve of the 13 patients. The 5-year progression-free survival rate was 63.5%.

**Fig 3:**
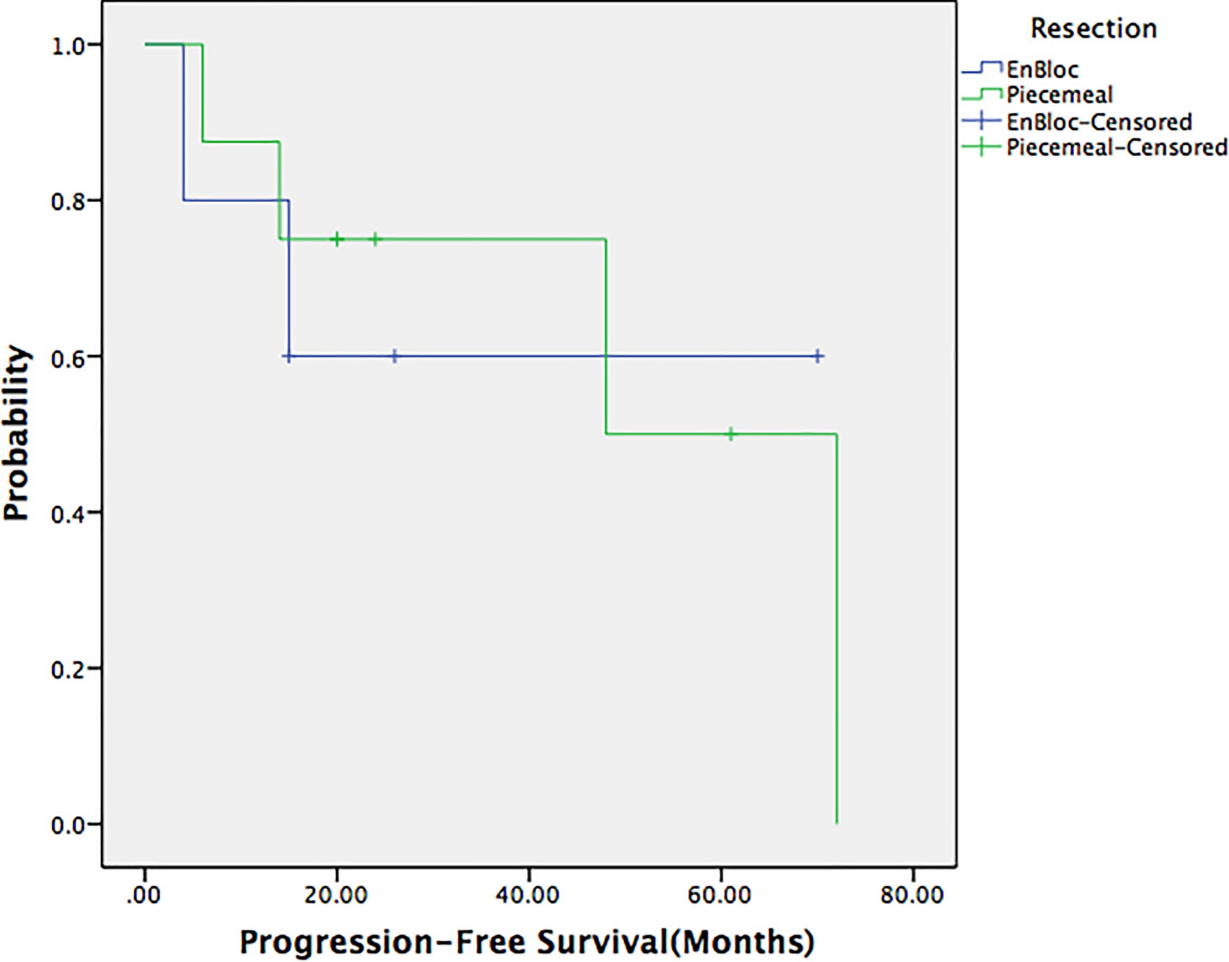
Progression-free survival curves of patients with en bloc resection and piecemeal resection. There was no significant difference between the two groups (*p* = 0.97).

A Chi-square test was used to compare the postoperative recurrence rate between the en bloc resection and the piecemeal resection groups (*p* = 0.68), and no significant difference was found between the benign and malignant solitary fibrous tumor groups (*p* = 0.44). The 5-year survival rate of the patients in this study was 83.3%, and the 5-year progression-free survival rate was 63.5%. The progression-free survival rates between the en bloc resection and piecemeal resection groups were also not significantly different (*p* = 0.97) (Figs 1–3).

In addition, 1 patient, whose initial symptom was recurrent episodes of hypoglycemia and syncope, also experienced weight gain, hand and foot thickening, clubbing, and changes in the texture of their finger (toe) nails and skin apart from the hypoglycemia. The patient was confirmed preoperatively to have malignant solitary fibrous tumors; prior to resection, after vascular embolization was performed, the patient’s paroxysmal hypoglycemia symptoms were relieved completely. One month after undergoing tumor resection using the posterior approach, the texture of the patient’s skin had improved, and 9 months after the surgery, the finger (toe) nails and skin had essentially returned to normal. Follow up was continued for 23 months after surgery, with no recurrence or metastasis of the tumor. The patient’s paroxysmal hypoglycemia symptoms were relieved completely.

**Fig 4:**
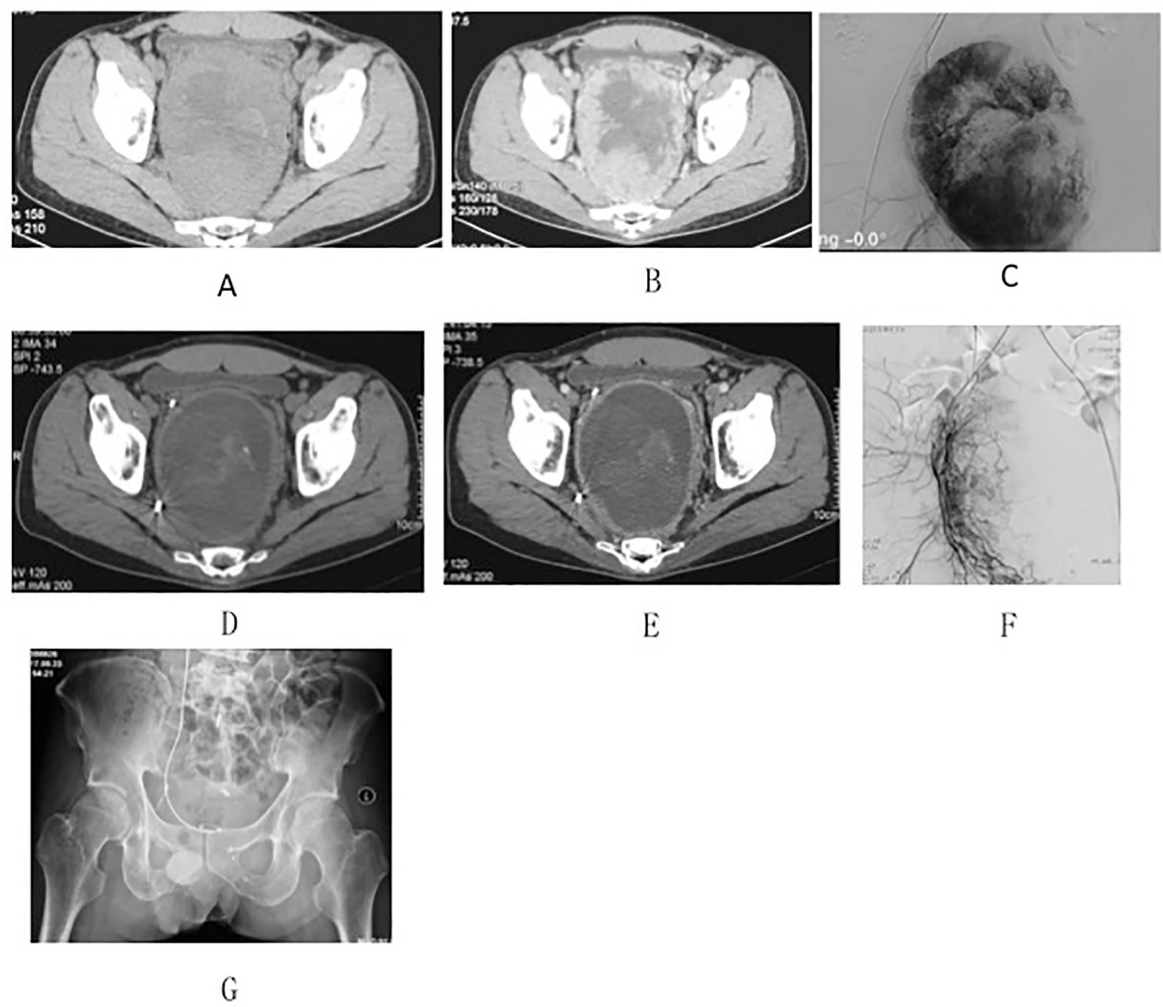
**(A-G) A 38-year-old male patient with sacral primary solitary fibrous tumor received oral administration of apatinib 750 mg qd treatment together with two courses of selective arterial embolization at our hospital, followed by surgical treatment.** Preoperative plain scan (A), enhanced CT (B), and angiography (C). The tumor was mainly solid component-based. Enhanced CT showed that the solid component was significantly enhanced, and angiography suggested that the tumor received an abundant blood supply. Plain scan (D), enhanced CT (E), and angiography (F) after treatment. The tumor size did not change significantly compared with that before treatment. However, the inside of the tumor mainly consisted of liquefied and necrotic regions, with solid components located only in the peripheral areas of the tumor. Enhanced CT showed only enhancement of the peripheral region. Angiography showed that the blood supply of the tumor had been reduced significantly. Postoperative pelvic anteroposterior X-ray film showing the low sacrococcygeal bone resection (G).

The tumor of the patient who received neoadjuvant apatinib therapy was significantly liquefied and necrotic, and the patient’s symptoms were relieved. The intraoperative bleeding volume of this patient was 600 ml, which was significantly less than the average bleeding volume (Fig 4A–4G).

## Discussion

### Clinical characteristics of solitary fibrous tumors

Solitary fibrous tumors can occur in various parts of the body, such as the thoracic cavity, the abdominal cavity, the pelvic cavity, the central nervous system, and the head, neck, and limbs. According to the data analysis of 804 patients over the past 40 years by the Surveillance, Epidemiology, and End Results (SEER) Program of the National Cancer Institute of the United States, patients with chest, abdominal, and pelvic cavity involvement accounted for 35.3% of cases, patients with central nervous system involvement accounted for 24.5%, and patients with head and neck or limb involvement accounted for 19.9% and 16.2%, respectively[6]. However, no previous study has reported cases of primary solitary fibrous tumors involving the pelvic ring.

The average onset age of patients in this group was 49.5 years. Although solitary fibrous tumors are typically found in patients 40–70 years of age, previous studies have reported onset ages ranging from 5–92 years of age[4,7]. For tumors involving the pelvic and abdominal cavities, because the tumor location is deep, the progression of the tumor is relatively slower; therefore, discovering these tumors at an early stage is difficult. For the patients in this study, by the time the tumor was clearly diagnosed, the tumor was relatively large, increasing the difficulty of surgical treatment. In previous literature, the survival rate of patients whose tumors originated from the pelvic and abdominal cavities and the retroperitoneal space was significantly lower than that of patients with solitary fibrous tumors in the limbs[8]. Some scholars have suggested that bone destruction by the tumor is an indication that the tumor is more invasive or more advanced[9]. Similar to this result, among the patients in this study, a total of 7 patients (53.8%) were diagnosed to have malignant solitary fibrous tumors. However, because the number of previously reported cases is very limited, the conclusion that bone destruction is significantly associated with the degree of malignancy of solitary fibrous tumors requires further confirmation.

Approximately 5% of patients with solitary fibrous tumors outside the pleura exhibit hypoglycemia, which may be caused by the large amount of insulin-like growth factor II (IGF II) produced by the tumor; such a manifestation is referred to as Doege-Potter syndrome[10], and 12–13% of patients with this syndrome suffer from malignant solitary fibrous tumors. Among the patients in this study, one patient had a malignant solitary fibrous tumor with initial symptoms of recurrent episodes of hypoglycemia and syncope. The follow-up was continued for 23 months after surgery, with no tumor recurrence or metastasis. The paroxysmal hypoglycemia, skin changes, and other concomitant endocrine symptoms were relieved completely.

### Surgical treatment of primary solitary fibrous tumors involving the pelvic ring

Most studies in the literature suggest that wide resection and en bloc resection are necessary for improving the postoperative local control rate and the overall survival of solitary fibrous tumors[3,5,11]. However, this study shows that piecemeal resection of the total tumor can also achieve good local control. There was no significant difference in the progression-free survival rates of the piecemeal resection group compared with the en bloc resection group.

One paper reviewed previous reports on 56 cases of spinal solitary fibrous tumors with follow-up results: among the 9 patients with subtotal resection, 5 experienced local recurrence in the 6-month to 21-year period following the surgery, and 1 had new spine lesions[12–18]. Among the 46 patients who received gross total resection, 41 (89.1%) did not experience recurrence[6,18–21]. For spinal solitary fibrous tumors, most tumors involved the dura mater, arachnoid tissue, and even the spinal cord[22–25], at which point wide resection and en bloc resection are often impossible. These studies reported that gross total resection can achieve good local control. Similarly, for sacral tumors, en bloc resection must also include resection of the sacral nerve, which will cause irreversible injuries to lower limb activity, urinary and bowel incontinence, and sexual dysfunction. Based on similar local control rates and more nerve root retention, the literature review and patient outcomes in this study, we postulate that for sacral solitary fibrous tumors, en bloc resection may not be necessary; the goal of the surgery is gross total resection while retaining the sacral nerves as much as possible. Therefore, a surgical method employing a simple posterior approach and piecemeal resection might be used for gross total resection of the tumor. This approach is similar to our previous surgical strategy for huge neurogenic sacral tumors[26].

For tumors involving the innominate bone, according to the region of the pelvic tumor, en bloc resection was mainly used for tumors involving region I or I+IV, and a pedicle screw and rod system was used for reconstructing the defect. For tumors involving region II, an internal hemipelvectomy method was frequently used. After resection of region II and region II+III tumors, an ordinary artificial hemipelvis should be used for reconstruction. There are three main reasons to use en bloc resection for tumors involving region II. First, the bone morphology around the acetabulum is irregular. If the curettage method is used, then the lesion may not be completely removed, and the presence of residual lesion and a subtotal resection are independent risk factors for recurrence. Second, if extensive curettage is performed to remove the lesion, significant damage to the acetabular structure may occur, making reconstruction of the hip joint difficult and affecting the postoperative function of the patient. Third, as this type of tumor often has an abundant blood supply, procedures performed on the lesion are often accompanied by continuous bleeding. En bloc resection can prevent excessive bleeding from the tumor. In addition, our previous experience confirmed that endohemipelvic resection and prosthetic hemipelvic replacement were safe and effective and that postoperative patient function was satisfactory[27–30]. Therefore, we used this surgical method to treat solitary fibrous tumors in region II.

Previous experience at our center has demonstrated that an abdominal aorta balloon block was more effective than these other approaches[31,32]. All patients in this study underwent preoperative embolization and/or abdominal aorta balloon block. Even after the procedures, the average intraoperative bleeding volume was still as high as 1757 ml. Therefore, for this type of tumor, adequate hemostasis and minimization of the operative time are required to ensure patient safety during surgery.

### Adjuvant therapy for solitary fibrous tumors

Studies on the effect of adjuvant therapy on solitary fibrous tumors are very limited. Previously published reports have primarily been case reports[33–35]. In recent years, targeted drug therapies for progressive solitary fibrous tumors have become a hotspot in adjuvant therapy for this tumor type. For non-case reports on the efficacy of targeted therapies on progressive solitary fibrous tumors according to RECIST and Choi criteria, see Table 2. Notably. the drugs mentioned in the reports are all multi-target drugs. In this study, we used apatinib to treat 1 patient with a huge sacral solitary fibrous tumor. In this study, our patient is the first clinically reported case in which apatinib was used to treat solitary fibrous tumors. After the treatment, the density inside the lesion was significantly reduced, and it was defined by the Choi criteria as PR. Thus, targeted treatment is a promising strategy in the treatment of this disease.

**Table 2:** Summary of outcomes of targeted drug treatment of progressive solitary fibrous tumors.

	RECIST				Choi			
	PR	SD	PD	Median PFS (months)	PR	SD	PD	Median PFS (months)
Bevacizumab + Temozolomide[36]	/	/	/	/	11/14	2/14	1/14	9.67
Sunitinib[37]	2/31	17/31	12/31	6	14/29	5/29	10/29	7
Sorafenib[38]	0	2/5	3/5	/	/	/	/	/
Pazopanib[39]	0	3/6	3/6	3	1/6	2/6	3/6	3
Trabectedin[40]	1/11	8/11	2/11	11.6	/	/	/	/
Dasatinib[41]	/	/	/	/	5/25	20/25		2

Of course, we realize that there are some limitations of this study: first, the number of cases is small, and it is a retrospective study; thus, a certain bias in the statistical analysis is inevitable. Furthermore, due to the limited sample size, performing multi-factor analysis is difficult. Defining the factors such as resection method, degree of malignancy that could affect the overall and event-free survival rates and the local control rate of patients still requires further study. In addition, only 1 death occurred, making it very difficult to analyze and compare the overall survival rate of this disease. Although this disease has a low incidence and cases that involve the pelvic ring are extremely rare, this study has the largest number of cases focusing on patients with this disease. As only 1 patient in this study was treated with neoadjuvant apatinib therapy and only 2 patients received radiotherapy after recurrence, accurately evaluating the efficacy of drug treatment, radiotherapy, and other adjuvant therapy strategies is difficult. However, for the first time, this study suggests that apatinib is effective against this disease—a conclusion that should be further studied. In future work, we will further investigate the above issues.

## Conclusion

The disease course of primary solitary fibrous tumors involving the pelvic ring is insidious. Consequently, the volume of the tumor is usually large at the time of diagnosis. These tumors have an abundant blood supply even when preoperative embolization and/or intraoperative abdominal aorta balloon block are implemented, which can result in a greater volume of intraoperative bleeding. If en bloc resection is not feasible, piecemeal resection can also achieve acceptable local control, particularly for patients with sacral tumors, as this approach can result in even better postoperative function than that achieved by other approaches. Recurrence and metastasis occur relatively late in the course of this disease; therefore, long-term follow-up is necessary.
